# Exposure to endocrine disruptors 17alpha-ethinylestradiol and estradiol influences cytochrome P450 1A1-mediated genotoxicity of benzo[*a*]pyrene and expression of this enzyme in rats

**DOI:** 10.1016/j.tox.2018.04.001

**Published:** 2018-05-01

**Authors:** Marie Stiborová, Helena Dračínská, Lucie Bořek-Dohalská, Zuzana Klusoňová, Jana Holecová, Markéta Martínková, Heinz H. Schmeiser, Volker M. Arlt

**Affiliations:** a Department of Biochemistry, Faculty of Science, Charles University, Albertov 2030, 128 40 Prague 2, Czech Republic; b Division of Radiopharmaceutical Chemistry, German Cancer Research Center (DKFZ), Im Neuenheimer Feld 280, 69120 Heidelberg, Germany; c Department of Analytical, Environmental and Forensic Sciences, MRC-PHE Centre for Environment and Health, King’s College London, 150 Stamford Street, London SE1 9NH, United Kingdom

**Keywords:** AhR, aryl hydrocarbon receptor, BaP, benzo[*a*]pyrene, BPDE, benzo[*a*]pyrene-7,8-dihydrodiol-9,10-epoxide, COMT, catechol-*O*-methyltransferase, CYP, cytochrome P450, EE2, 17α-ethinylestradiol, DMSO, dimethylsulfoxide, ED, endocrine disruptor, dG-*N*^2^-BPDE, 10-(deoxyguanosin-*N*^2^-yl)-7,8,9-trihydroxy-7,8,9,10-tetrahydrobenzo[*a*]pyrene, EROD, 7-ethoxyresorufin *O*-deethylation, GAPDH, glyceraldehyde 3-phosphate dehydrogenase, NADPH, nicotinamide adenine dinucleotide reduced, *m*EH, microsomal epoxide hydrolase, NQO1, NAD(P)H:quinone oxidoreductase 1, PAH, polycyclic aromatic hydrocarbon, POR, NADPH:CYP reductase, TLC, thin layer chromatography, RAL, relative adduct labelling, Endocrine disruptors, 17alpha-ethinylestradiol, Estradiol, Benzo[*a*]pyrene, Cytochrome P450, DNA-adducts

## Abstract

Endocrine disruptors (EDs) are compounds that interfere with the balance of the endocrine system by mimicking or antagonising the effects of endogenous hormones, by altering the synthesis and metabolism of natural hormones, or by modifying hormone receptor levels. The synthetic estrogen 17α-ethinylestradiol (EE2) and the environmental carcinogen benzo[*a*]pyrene (BaP) are exogenous EDs whereas the estrogenic hormone 17β-estradiol is a natural endogenous ED. Although the biological effects of these individual EDs have partially been studied previously, their toxicity when acting in combination has not yet been investigated. Here we treated Wistar rats with BaP, EE2 and estradiol alone or in combination and studied the influence of EE2 and estradiol on: (*i*) the expression of cytochrome P450 (CYP) 1A1 and 1B1 in rat liver on the transcriptional and translational levels; (*ii*) the inducibility of these CYP enzymes by BaP in this rat organ; (*iii*) the formation of BaP-DNA adducts in rat liver *in vivo*; and (*iv*) the generation of BaP-induced DNA adducts after activation of BaP with hepatic microsomes of rats exposed to BaP, EE2 and estradiol and with recombinant rat CYP1A1 *in vitro*. BaP acted as a strong and moderate inducer of CYP1A1 and 1B1 in rat liver, respectively, whereas EE2 or estradiol alone had no effect on the expression of these enzymes. However, when EE2 was administered to rats together with BaP, it significantly decreased the potency of BaP to induce *CYP1A1* and *1B1* gene expression. For EE2, but not estradiol, this also correlated with a reduction of BaP-induced CYP1A1 enzyme activity in rat hepatic microsomes. Further, while EE2 and estradiol did not form covalent adducts with DNA, they affected BaP-derived DNA adduct formations *in vivo* and *in vitro*. The observed decrease in BaP-DNA adduct levels in rat liver *in vivo* resulted from the inhibition of CYP1A1-mediated BaP bioactivation by EE2 and estradiol. Our results indicate that BaP genotoxicity mediated through its activation by CYP1A1 in rats *in vivo* is modulated by estradiol and its synthetic derivative EE2.

## Highlights

• 17α-ethinylestradiol **(**EE2) and estradiol affect genotoxicity of benzo[*a*]pyrene (BaP) in rats.
• Cytochrome P450 (CYP) 1A1 and 1B1 are induced in rats by BaP but not EE2 and estradiol.
• Exposure of rats to EE2, estradiol and BaP decreased BaP-DNA adduct formation *in vivo*.
• The decrease results from inhibition of CYP1A1-mediated BaP activation by EE2 and estradiol.

## Graphical abstract

Effects of 17α-ethinylestradiol (EE2) and estradiol (ESTRA) on DNA adducts formed by benzo[*a*]pyrene (BaP).

**Figure:**
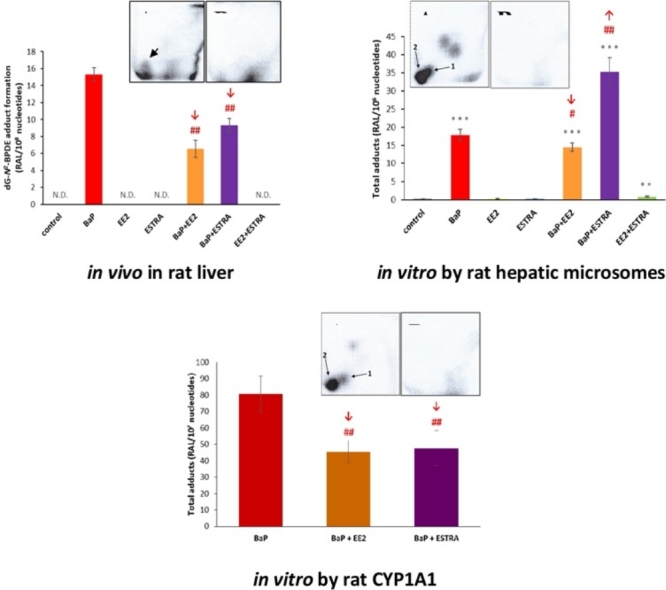

## 1 Introduction

The term “endocrine disruptor” (ED) is used for compounds that mimic or antagonise the effects of endogenous hormones, alter the synthesis and metabolism of natural hormones or modify hormone receptor levels. The synthetic estrogen 17α-ethinylestradiol (EE2) and the carcinogenic environmental pollutant benzo[*a*]pyrene (BaP) belong to a group of chemicals assigned as exogenous endocrine disruptive compounds while the estrogenic hormone estradiol, or more precisely, 17β-estradiol, is a natural endogenous ED. The biological effects of these EDs depend on their metabolism. Although the toxic effects of these EDs are partially known, apart from BaP, information on their genotoxic and carcinogenic properties mediated during metabolism is scarce.

**Fig. 1:**
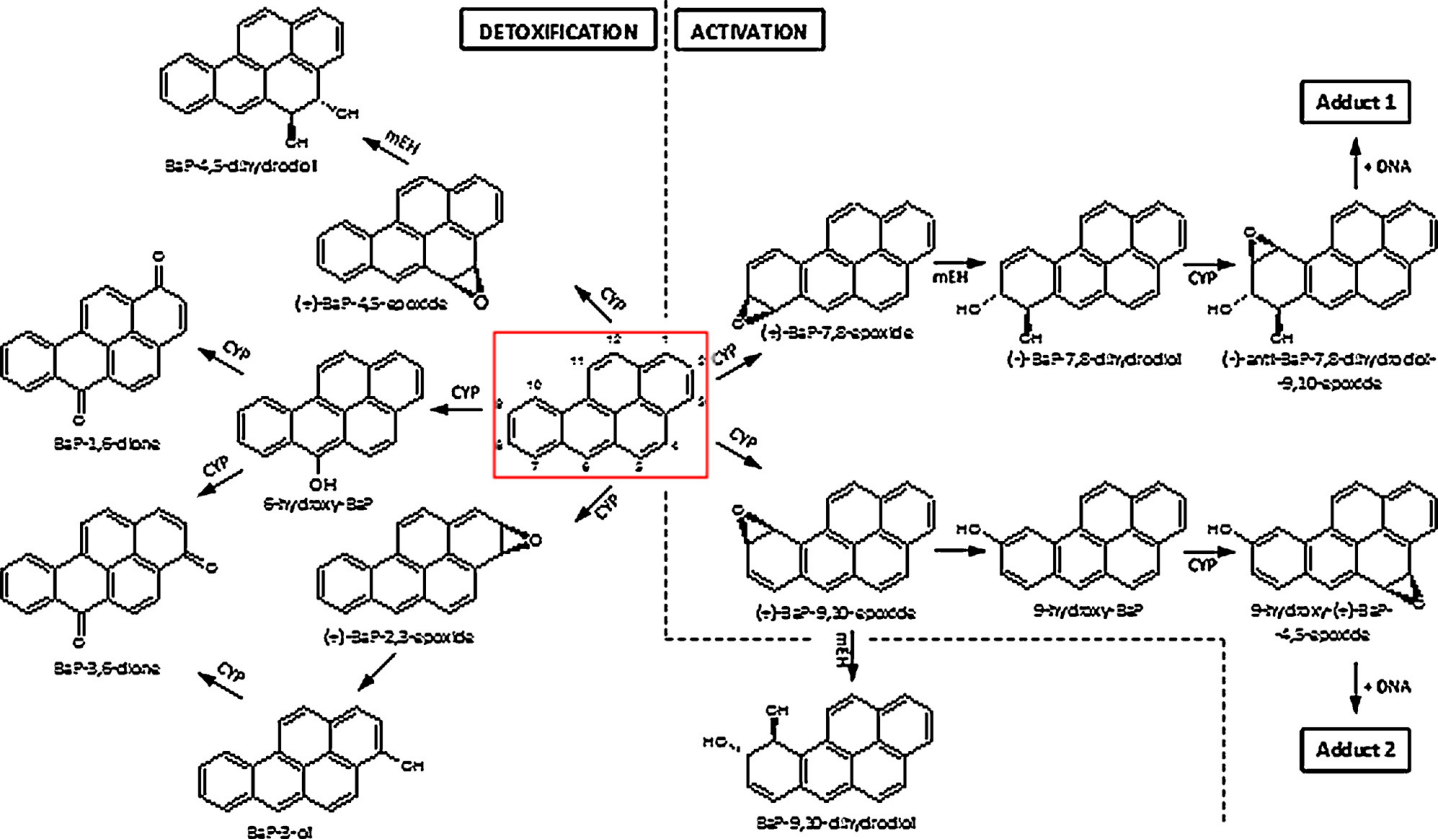
Proposed pathways of biotransformation and DNA adduct formation of BaP catalysed by CYP enzymes and *m*EH. The typical three-step activation process by CYPs followed by hydrolysis by *m*EH leads to BPDE which forms dG-*N*^2^-BPDE (adduct 1) and the two-step activation process by CYP leads to the formation of 9-hydroxy-BaP-4,5-epoxide that can react with deoxyguanosine in DNA (adduct 2). Formation of BaP detoxification metabolites are shown in the left part of the figure.

BaP is a polycyclic aromatic hydrocarbon (PAH) that has been classified as human carcinogen (Group 1) by the International Agency for Research on Cancer (IARC) (IARC, 2010). BaP and other PAHs are produced mainly by incomplete combustion of organic matter. Their ubiquitous presence in the environment leads to measurable background levels of exposure in the general population (IARC, 2010). Beside the inhalation of polluted air, the main sources of exposure are tobacco smoke and diet (Baird et al., 2005). BaP has been shown to cause cytotoxic, genotoxic, neurotoxic, mutagenic and carcinogenic effects in various tissues and cell types (Siddens et al., 2012; Wohak et al., 2016; Krais et al., 2016; Long et al., 2016, Long et al., 2017; Chepelev et al., 2016). BaP requires metabolic activation prior to reaction with DNA (Reed et al., 2018). Cytochrome P450 (CYP) enzymes, mainly CYP1A1 and 1B1, are the most important enzymes involved in this process, in combination with microsomal epoxide hydrolase (*m*EH) (Fig. 1) (Nebert et al., 2013; Arlt et al., 2015; Stiborová et al., 2014, Stiborová et al., 2016a, Stiborová et al., 2016b). First, CYP1A1 enzyme oxidises BaP to an epoxide that is then converted to a dihydrodiol by *m*EH (*i.e.* BaP-7,8-dihydrodiol). Further bioactivation by CYP1A1 leads to the ultimately reactive species, BaP-7,8-dihydrodiol-9,10-epoxide (BPDE) that can react with DNA, forming adducts preferentially at guanine residues (Fig. 1). The 10-(deoxyguanosin-*N*^2^-yl)-7,8,9-trihydroxy-7,8,9,10-tetrahydrobenzo[*a*]pyrene (dG-*N*^2^-BPDE) adduct is the major product of the reaction of BPDE with DNA *in vivo* (Arlt et al., 2008, Arlt et al., 2012) and preferentially leads to the induction of G to T transversion mutations (Alexandrov et al., 2016; Kucab et al., 2015; Nik-Zainal et al., 2015). Alternatively, BaP-7,8-dihydrodiol can be activated by aldo-keto reductases leading to BaP-7,8-dione which is also capable of forming DNA adducts and generating oxidative damage to DNA (Penning, 2014). However, BaP is also oxidised to other metabolites such as other dihydrodiols, BaP-diones and further hydroxylated metabolites (Indra et al., 2013, Indra et al., 2014; Stiborová et al., 2014, Stiborová et al., 2016a, Stiborová et al., 2016b; Sulc et al., 2016). Although most of these metabolites are detoxification products, BaP-9-ol (9-hydroxy-BaP) is the precursor of 9-hydroxy-BaP-4,5-epoxide that can form another adduct with deoxyguanosine in DNA (Fig. 1). Expression of CYP enzymes of the family 1 (CYP1A1 and 1B1), which predominantly metabolise BaP, are known to be up-regulated by the aryl hydrocarbon receptor (AhR); BaP itself can bind to and activate AhR thereby enhancing its own metabolic activation (Hockley et al., 2007, Hockley et al., 2008).

**Fig. 2:**
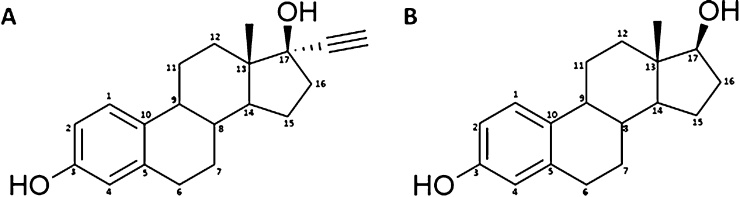
Structures of EE2 and estradiol.

Synthetic estrogen EE2 (Fig. 2) is widely used as a major component in oral contraceptives (Bolt, 1979). Incorporation of an acetylenic moiety into the estradiol molecule resulted in increased oral availability of the drug. Although there is evidence for carcinogenicity of EE2 in experimental animals (IARC, 1979), reports on the genotoxic potential of this ED are contradictory (Siddique et al., 2005). EE2 is metabolised by hydroxylation at the 2, 4, 6, and 16α position of the steroid nucleus (Back et al., 1984; Rogers et al., 1987; Stanczyk et al., 2013; Zhang et al., 2007). The 2-hydroxy-EE2 derivative can subsequently be methylated *in vivo* to give 2-methoxyethinylestradiol (Back et al., 1984; Rogers et al., 1987). The CYP enzymes predominantly catalysing the 2-hydroxylation of EE2 in human liver microsomes are CYP2C9 and 3A4, whereas CYP2C8, 2C19, and 1A2 only contribute to a lesser extent to this reaction. EE2 is also a substrate of various rat hepatic CYPs. Rat CYP2C6 and 2C11 are most efficient in catalysing the formation of the major EE2 metabolite 2-hydroxy-EE2, whereas EE2 hydroxylation by rat CYP2A and 3A predominantly leads to a minor hydroxylation metabolite, whose structure remains to be identified (Borek-Dohalska et al., 2014, Borek-Dohalska et al., 2015).

Metabolism of the estrogenic hormone estradiol (Fig. 2) has been extensively studied in a large number of studies. It can act as a weak carcinogen and weak mutagen capable of inducing genetic lesions (Liehr, 2000). Estradiol undergoes extensive oxidative metabolism at various positions leading to the formation of various hydroxylated or keto metabolites. This oxidative metabolism is catalysed by several CYPs present in liver and in extrahepatic estrogen target organs, including enzymes of the CYP1, CYP3A and CYP2C subfamilies (reviewed in Zhu and Lee, 2005). Aromatic hydroxylation at either the C2 or C4 position is the major route of estradiol metabolism in humans and other mammals, although there is less 4-hydroxylation than 2-hydroxylation. 2-Hydroxyestradiol is considered as a non-toxic metabolite, whereas 4-hydroxyestradiol, which is primarily formed by the extrahepatic CYP1B1, is known to be genotoxic (Lee et al., 2003). Several CYPs including CYPs of the subfamily 1A, CYP1B1, CYPs of the subfamily 2C, CYPs of the subfamily 3A and CYP2D6 were shown to catalyse the hydroxylation of estradiol to 2-hydroxyestradiol and/or 4-hydroxyestradiol. CYP1A2 and 3A4 also catalyse the 16α-hydroxylation of estradiol to estriol (Badawi et al., 2001).

Although the biological effects caused by individual EDs have partially been investigated, their combined impact has essentially not been studied. Therefore, the aim of the present study was to investigate the effect of EE2 and estradiol on the CYP-mediated genotoxicity of BaP. Male Wistar rats were used as animal model and the formation of covalent BaP-derived DNA adducts was studied *in vivo* in the liver and in *in vitro* incubations using hepatic microsomes of rats exposed to EDs. Complementary *in vitro* studies used recombinant rat CYP1A1 in Supersomes™. Besides studying BaP-DNA adduct formation by ^32^P-postlabelling, we examined the influence of EE2 and estradiol on expression of major CYP enzymes (CYP1A1 and 1B1) catalysing BaP activation using qPCR and Western blotting. Livers of male rats were utilised because liver tissue contains most biotransformation enzymes (e.g. CYPs) known to activate BaP by oxidition and previous studies have shown that these enzymes can also be induced in rat liver (Hodek et al., 2013), thereby modulating the genotoxicity (i.e. DNA adduct formation) of this carcinogen. In our experiments, rats were treated with BaP, EE2 and estradiol alone or in combinations and livers of these animals were analysed for these effects.

## 2 Materials and methods

### 2.1 Chemicals and enzymes

17a-ethinylestradiol (EE2), glucose-6-phosphate, NADP^+^, NADPH, 17β-estradiol, 7-ethoxyresorufin, benzo[*a*]pyrene (BaP) were obtained from Sigma Chemical Co. (St. Louis, MO, USA). Sudan I was purchased from BDH (Poole, UK), glucose-6-phosphate dehydrogenase from Serva (Heidelberg, Germany) and bicinchoninic acid from Pierce (Rockford, IL, USA). All other chemicals were of analytical purity or better. Rat CYP1A1-Supersomes™, microsomes isolated from insect cells transfected with a baculovirus construct containing cDNA of recombinant rat CYP1A1 and NADPH:CYP reductase (POR), were purchased from Gentest Corp. (Woburn, MI, USA).

### 2.2 Treatment of rats

All animal experiments were conducted in accordance with the Regulations for the Care and Use of Laboratory Animals (311/1997, Ministry of Agriculture, Czech Republic), which is in compliance with the Declaration of Helsinki. Male Wistar rats (150 g, AnLab, Czech Republic), were housed in groups of 3 in wire cages at 22 °C with a 12 h light/dark period and *ad libitum* diet (ST-1 diet from Velaz, Czech Republic) and water access.

Rats were divided into seven groups (*n* = 3/group). Three groups were treated by oral gavage with one dose of either BaP (150 mg/kg body weight [bw]), estradiol (20 mg/kg bw) or EE2 (20 mg/kg bw). The next three groups were treated once with combinations of BaP with EE2, BaP with estradiol or EE2 with estradiol. Test compounds were all dissolved in sunflower oil. The control group received sunflower oil only. All animals were sacrificed after 48 h and liver tissues were snap-frozen in liquid nitrogen and stored at −80 °C until further analysis.

### 2.3 BaP-DNA adduct detection by ^32^P-postlabelling analysis

Genomic DNA from whole liver tissue was isolated by a standard phenol-chloroform extraction method and DNA adducts were measured for each DNA sample using the nuclease P1 enrichment version of the thin-layer chromatography (TLC)-^32^P-postlabelling method as described previously (Arlt et al., 2008). After chromatography TLC plates were scanned using a Packard Instant Imager (Downers Grove, IL, USA). DNA adduct levels were calculated as described (Schmeiser et al., 2013). Results were expressed as relative adduct labelling (RAL).

### 2.4 Preparation of microsomes

Hepatic microsomes from all groups of rats were isolated as described previously (Arlt et al., 2008; Stiborová et al., 2013). Microsomes were isolated from 3 pooled livers of rats of each treatment group. Protein concentration in the microsomal fraction was measured using the bicinchoninic acid protein assay with bovine serum albumin as standard. Pooled microsomal fractions were used for further experiments.

### 2.5 Western blot analysis

For the detection of individual CYP enzymes, 75 μg of microsomal protein was separated via sodium dodecylsulfate polyacrylamide gele electrophoresis (SDS-PAGE) (10% acrylamide, Bio-Rad). The polyvinylidene fluoride (PVDF) membrane after the electrotransfer was blocked in a solution of 5% skim milk in TBST-Tween buffer (20 mM Tris/HCl, 150 mM NaCl, 0.1% Tween 20, pH 7.5) for 1 h at room temperature. The CYP1A1 was detected with a rabbit anti-rat CYP1A1 primary antibody (BioTech, Czech Republic) (dilution 1:2500) and CYP1B1 with a rabbit anti-rat CYP1B1 primary antibody (Santa Cruz Biotechnology, USA) (dilution 1:400) diluted in 5% skim milk in Tris-buffered saline with Tween 20 (TBST-Tween buffer) over night at 4 °C. After washing in TBST-Tween buffer, membrane was incubated with alkaline phosphatase-conjugated rabbit IgG anti-rabbit IgG in 5% skim milk in TBST-Tween buffer (dilution 1:1430) for 1 h at room temperature. Protein bands were visualized with the alkaline phosphatase substrate, 5-bromo-4-chloro 3- indolyl phosphate/nitro blue tetrazolium tablet. To assure comparable protein amount and expression, we routinely use anti-glyceraldehyde 3-phosphate dehydrogenase (GAPDH) for normalisation of the Western blot data.

### 2.6 CYP1A enzyme activity assays

The rat hepatic microsomal fractions were characterised for CYP1A1 enzyme activity using Sudan I hydroxylation (Stiborová et al., 2002, Stiborová et al., 2005) and for CYP1A enzyme activities we used 7-ethoxyresorufin *O*-deethylation (EROD) (Stiborová et al., 2002, Stiborová et al., 2005).

### 2.7 CYP1A1 and 1B1 mRNA content in rat livers

Total RNA was isolated from frozen livers of all rat groups and mRNA quantified by RT-PCR exactly as described (Stiborová et al., 2008).

### 2.8 Microsomal incubations for BaP-DNA adduct formation

Incubation mixtures consisted of 50 mM potassium phosphate buffer (pH 7.4). 1 mM reduced nicotinamide adenine dinucleotide (NADPH), pooled hepatic microsomal fraction (0.5 mg/ml protein) from all treatment groups, 0.1 mM BaP (dissolved in 7.5 μl dimethylsulfoxide [DMSO]) and calf thymus DNA (0.5 mg) in a final volume of 750 μl. Incubations were carried out at 37 °C for 90 min (Arlt et al., 2008). Control incubations were carried out: (*i*) without microsomes; (*ii*) without NADPH; (*iii*) without DNA; and (*iv*) without BaP. After incubation, DNA was isolated by a standard phenol-chloroform extraction method. BaP-DNA adduct formation was determined by ^32^P-postlabelling as described above.

### 2.9 Determination of BaP-DNA adduct formation catalysed by recombinant rat CYP1A1 and the effects of EE2 and estradiol on this process

Incubation mixtures used for studying BaP activation to species forming BaP-DNA adducts by recombinant rat CYP1A1 in Supersomes™ contained in a final volume of 750 μl, 50 mM potassium phosphate buffer (pH 7.4), 0.1 mM BaP (dissolved in 7.5 μl DMSO), calf thymus DNA (0.5 mg) and 100 nM rat recombinant CYP1A1 in Supersomes™ (in combination with its reductase POR) in the presence or absence of 0.1 mM EE2 or estradiol (both dissolved in 7.5 μl DMSO). Control incubations were carried out: (*i*) without CYP1A1-Supersomes™^M^; (*ii*) without NADPH; (*iii*) without DNA; and (*iv*) without BaP. After the incubation DNA was isolated by a standard phenol-chloroform extraction method. BaP-DNA adduct formation was determined by ^32^P-postlabelling as described above.

## 3 Results

### 3.1 Effect of EE2 or estradiol on BaP-DNA adduct formation *in vivo* in rats

**Fig. 3:**
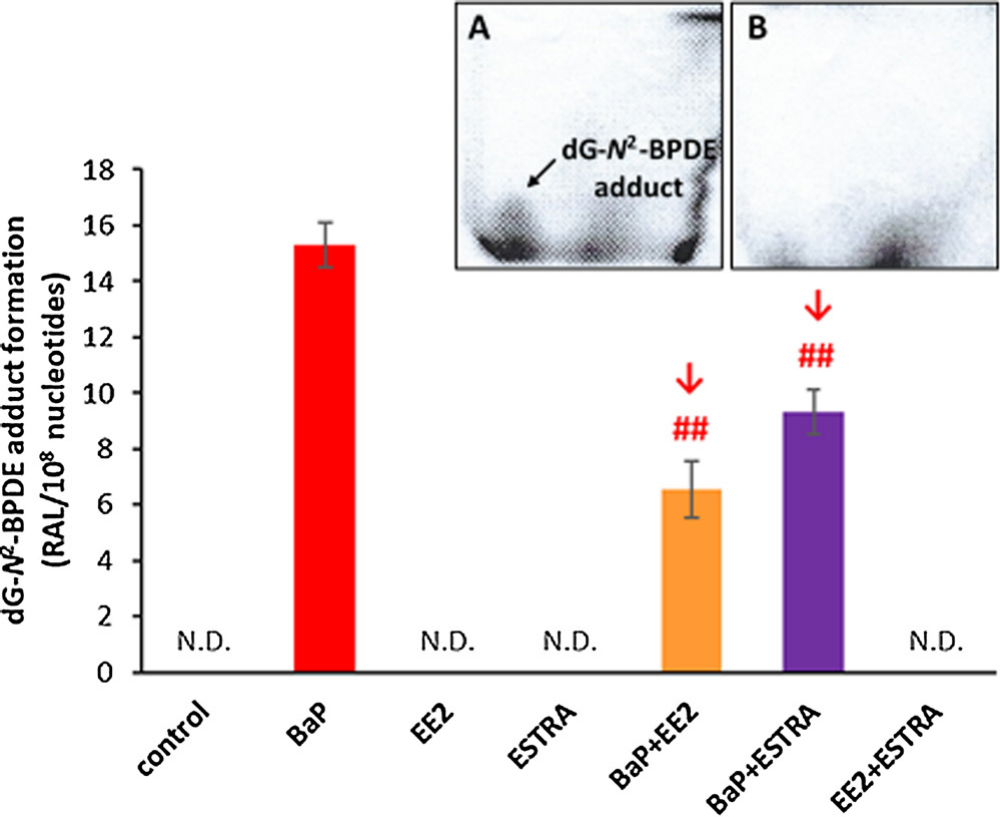
DNA adduct formation by BaP (the dG-*N^2^*-BPDE adduct), measured by TLC-^32^P-postlabelling, in livers of rats treated with BaP, EE2 or estradiol (ESTRA) alone and in combination (BaP + EE2, BaP + ESTRA or EE2 + ESTRA). *Insert A and B*: Autoradiographic profiles of DNA adducts formed in liver of BaP-treated rats (A) and those treated with vehicle only (control) (B). Values represent mean total RAL (relative adduct labelling) ± SD (*n* = 3; analyses of three hepatic samples). N.D., not detected. Statistical analysis was performed by ANOVA with post-hoc Tukey HSD Test. ^##^*P <* 0.01 significant differences between levels of dG-*N^2^*-BPDE adducts in liver of rats treated with BaP alone and with combination of BaP with EE2 or estradiol (ESTRA).

Covalent DNA adduct formation was determined by ^32^P-postlabelling in the livers of male Wistar rats treated with BaP alone or in combination with EE2 or estradiol. Using the nuclease P1 enrichment version of the assay, DNA adducts were found in all liver samples from rats treated with BaP, BaP/EE2 and BaP/estradiol. The BaP-DNA adduct pattern obtained *in vivo* consisted of one major adduct spot (assigned adduct spot 1) (Fig. 3, insert A), which was previously identified as 10-(deoxyguanosin-*N*^2^-yl)-7,8,9-trihydroxy-7,8,9,10-tetrahydrobenzo[*a*]pyrene (dG-*N^2^*-BPDE) (Arlt et al., 2008). No DNA adduct were detected in livers of control rats (Fig. 3, insert B), rats treated with EE2 or estradiol alone or rats treated with a combination of both EE2 and estradiol (data not shown).

In livers of rats treated with BaP together with EE2 or estradiol, the levels of dG-*N^2^*-BPDE adduct were 2.3-fold and 1.6-fold lower than those in rats exposed to BaP alone, respectively (Fig. 3). Therefore, EE2 and estradiol, when administered to rats together with BaP, modulate the metabolic pathway of BaP consequently leading to a decrease in BaP-derived DNA adduct formation in this rat organ.

### 3.2 Effect of EE2 or estradiol on BaP-DNA adduct formation using rat hepatic microsomes *in vitro*

**Fig. 4:**
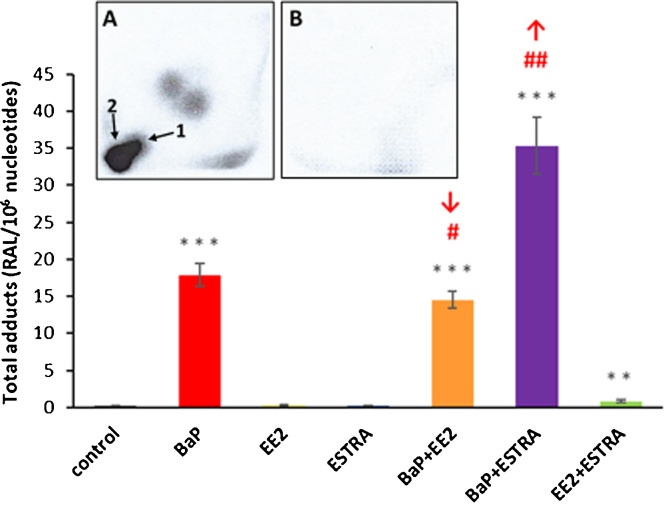
DNA adduct formation by BaP, measured by TLC-^32^P-postlabelling, activated with hepatic microsomes isolated from livers of rats exposed to BaP, EE2, estradiol (ESTRA) alone and in combination (BaP+EE2, BaP+ESTRA or EE2+ESTRA). *Insert A and B*: Autoradiographic profile of DNA adducts formed in incubations of BaP and DNA with hepatic microsomes of rats treated with BaP (A) and that with the same microsomes but without BaP (B). Values represent mean total RAL (relative adduct labelling) ± SD (*n *= 3; analyses of three independent *in vitro* incubations). ***P < 0.001 (ANOVA with post-hoc Tukey HSD Test), levels of total BaP-adducts formed by incubations of BaP and DNA with hepatic microsomes of rats treated with tested EDs significantly different from incubations with microsomes of control rats (treated with vehicle only). ^##^*P *< 0.01, ^#^*P *< 0.05 (ANOVA with post-hoc Tukey HSD Test), significant differences between levels of total DNA-BaP adducts formed in incubations with hepatic microsomes of rats treated with BaP alone and combinations of BaP with EE2 and estradiol (ESTRA).

Next we investigated the ability of hepatic microsomes isolated from treated and control rats to catalyse BaP-DNA adduct formation *in vitro*. The BaP-DNA adduct pattern obtained by ^32^P-postlabelling analysis in *in-vitro* microsomal incubations consisted of up to two major adduct spots (Fig. 4, insert A). Adduct spot 1 was identified to correspond to the dG-*N^2^*-BPDE adduct (Arlt et al., 2008) and was also generated in rat livers *in vivo* (compare Fig. 3). The other major spot detected by TLC (assigned adduct 2) has not yet been fully structurally identified, but is likely to be derived from the reaction of 9-hydroxy-BaP-4,5-epoxide with deoxyguanine in DNA (see Fig. 1) (Schoket et al., 1989; Nesnow et al., 1993; Fang et al., 2001). The biotransformation pathways leading to the formation of both these adducts are illustrated in Fig. 1. The dG-*N^2^*-BPDE adduct, which was the only adduct detected *in vivo*, was formed by microsomes at lower amounts than the 9-hydroxy-BaP-4,5-epoxide-derived adduct (adduct 2). Additionally two minor adduct spots were also visible by autoradiography (see Fig. 4, insert A) and it has been suggested previously that they also can be BaP-derived DNA adducts (Schoket et al., 1989; Fang et al., 2001). Indeed, these minor adducts were not found in control incubations carried out without BaP (Fig. 4, insert B). As the origin of these adducts is currently unknown their levels were not quantified in the present study.

Whereas treatment of rats to BaP together with EE2 decreased the efficiency of microsomes to form BaP-DNA adducts *in vitro*, which was analogous to the process found *in vivo*, treatment of rats with BaP in combination with estradiol actually stimulated the enzymatic efficacy of hepatic microsomes to activate BaP *in vitro*, leading to a ∼2-fold increase in BaP-DNA adduct levels (Fig. 4 & Supplementary Table S1). This finding is opposite to the results found *in vivo* where treatment of rats with BaP together with estradiol inhibited BaP-DNA adduct formation in the livers (see Fig. 3). No BaP-DNA adducts were formed in control incubations with microsomes of all treatment groups without BaP (see Fig. 4, insert B, showing the autoradiography of DNA isolated from incubation of DNA with microsomes of BaP-exposed rats but without the addition of BaP *in vitro*) or with BaP but without microsomes (data not shown).

Because CYP1A1 and 1B1 enzymes activate BaP to metabolites capable of forming DNA adducts (Stiborová et al., 2014, Stiborová et al., 2016a; Wohak et al., 2016; Krais et al., 2016; Sulc et al., 2016), their expression might determine the levels of BaP-DNA adducts. Therefore, we investigated the expression of CYP1A1 and 1B1 in hepatic microsomes and the impact of EE2 and estradiol on their expression.

### 3.3 Effect of EE2 or estradiol on BaP-induced expression of CYP1A1 and 1B1 in rat hepatic microsomes

**Fig. 5:**
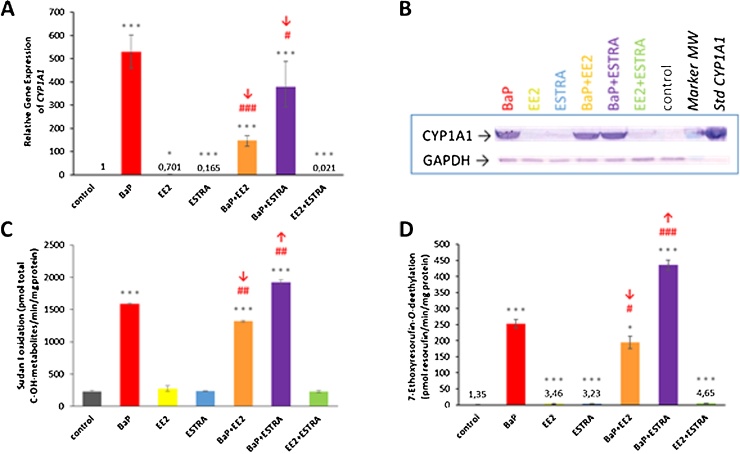
Relative *CYP1A1* gene expression in rat liver tissue (A), Western blot analysis of CYP1A1 (B) and its marker activities Sudan I hydroxylation (C) and EROD (D) in hepatic microsomes. GAPDH protein expression was used as a loading control. Representative image of the Western blotting is shown, and at least triplicate analysis was performed in separate experiments. ***P < 0.001; **P < 0.01; *P < 0.05; (ANOVA with post-hoc Tukey HSD Test), levels of data analysed in liver (qPCR) and hepatic microsomes (CYP1A1 marker activities) of rats treated with EDs tested in combination with BaP significantly different from control rats (treated with vehicle only). ^###^*P <* 0.001; ^##^*P <* 0.01; ^#^*P <* 0.05 (ANOVA with post-hoc Tukey HSD Test), significant differences between levels of data in livers (qPCR) and hepatic microsomes of rats treated with BaP and tested EDs significantly different from rat treated with BaP alone.

**Fig. 6:**
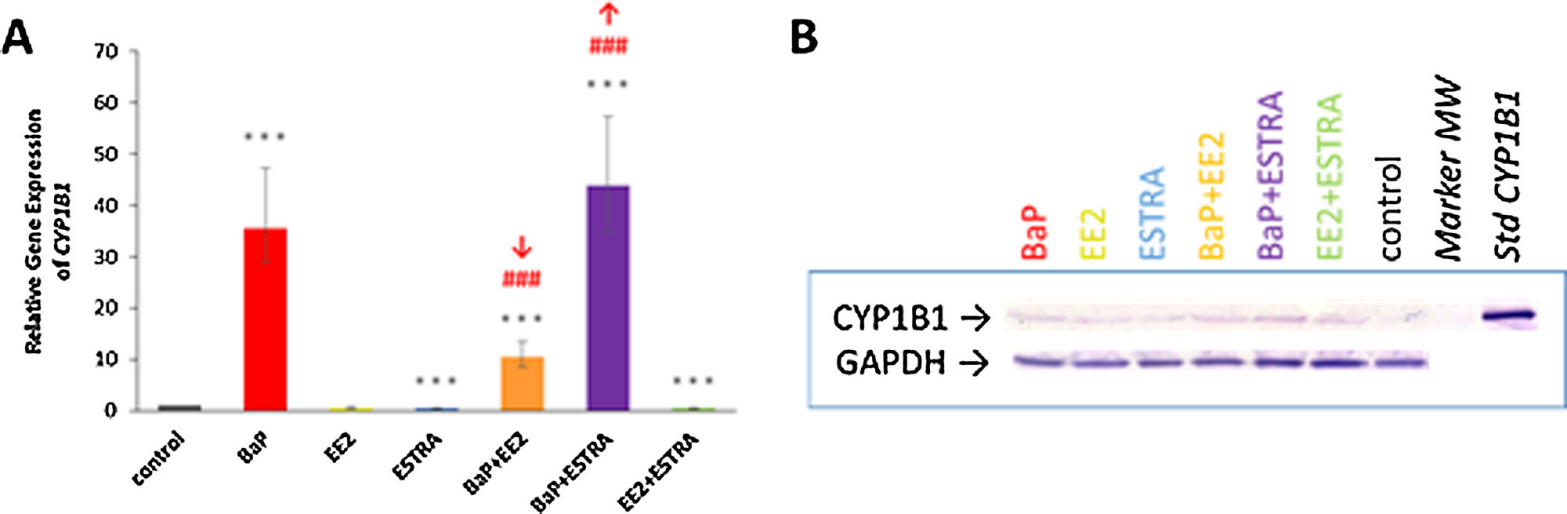
Relative *CYP1B1* gene expression in rat liver tissue (A) and Western blot analysis of CYP1B1 in hepatic microsomes (B). GAPDH protein expression was used as a loading control. Representative image of the Western blotting is shown, and at least triplicate analysis was performed in separate experiments. ***P < 0.001 (ANOVA with post-hoc Tukey HSD Test), levels of data analysed measured in liver (qPCR) of rats treated with tested EDs together with BaP significantly different from control rats (treated with vehicle only). ^###^*P <* 0.001 (ANOVA with post-hoc Tukey HSD Test), significant differences between levels of data in livers (qPCR) treated with BaP and tested EDs with BaP significantly different from rat treated with BaP alone.

As shown in Fig. 5, Fig. 6, BaP acts as a strong and moderate inducer of CYP1A1 and 1B1, respectively, both on the transcriptional and translational levels, whereas EE2, estradiol or their combinations had no induction effect. However, when the estrogenic compounds (EE2, estradiol) were administered to rats together with BaP, they affected the degree of BaP-mediated CYP1A1 and 1B1 induction in rat livers on the transcriptional level (Figs. 5A and 6A).

As shown in Fig. 5, BaP treatment of rats together with EE2 decreased BaP-induced expression of *CYP1A1*, CYP1A1 protein levels and Sudan I hydroxylation, a marker for CYP1A1 enzyme activity (Stiborová et al., 2002, Stiborová et al., 2005). For treatment of BaP with estradiol, BaP-induced *CYP1A1* gene expression was also decreased, but virtually no effect was observed on the protein level (Fig. 5A and B). Furthermore, Sudan I hydroxylation was slightly increased (up to 1.2-fold; *P <* 0.01) compared to CYP1A1 enzyme activity in hepatic microsomes of rats treated with BaP alone (Fig. 5C). Similar trends where observed for both EE2 and estradiol when *O*-deethylation of 7-ethoxyresorufin (EROD), another marker for CYP1 enzyme activity, was determined (Fig. 5D). *O*-deethylation of 7-ethoxyresorufin reaction is mainly catalysed by CYP1A1, but it can also be mediated by CYP1B1. Nevertheless, the efficiency of CYP1B1 to catalyse this reaction is only ∼2% of the efficacy of the CYP1A1 enzyme (Henderson et al., 2000).

CYP1B1 protein levels were also induced in livers of BaP-treated rats, but induction was much lower compared to CYP1A1 (compare Fig. 5, Fig. 6). In contrast, treatment with EE2 and/or estradiol essentially showed no effect Fig. 6). Exposure of rats to BaP/EE2 lowered BaP-induced *CYP1B1* gene expression, but this was not reflected on the protein level. On the contrary, BaP/estradiol exposure slightly elevated BaP-induced *CYP1B1* gene expression (Fig. 6A) and this effect was also seen on the protein level (Fig. 6B). For CYP1B1 no enzymatic activity could be analysed because a specific marker substrate of this enzyme has not been identified as yet (Henderson et al., 2000; Nishida et al., 2013; Wang et al., 2016).

### 3.4 Effect of EE2 or estradiol on BaP-DNA adduct formation catalysed by rat recombinant CYP1A1

**Fig. 7:**
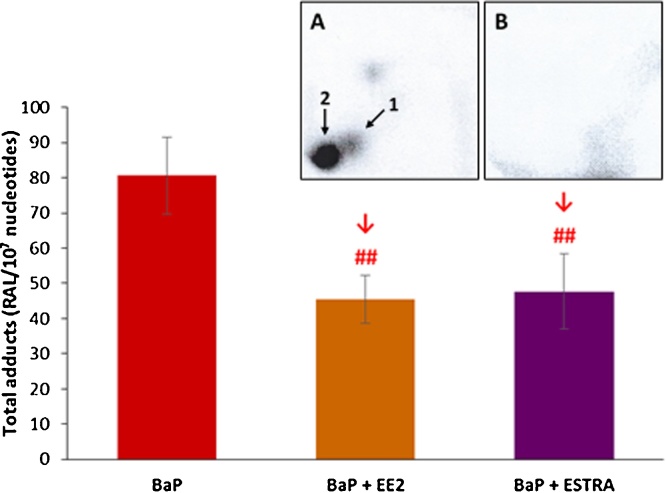
DNA adduct formation by BaP, measured by TLC-^32^P-postlabelling, activated with recombinant rat CYP1A1 expressed in Supersomes™ *in vitro* and the effect of EE2 and estradiol (ESTRA) on BaP-DNA adduct levels. *Insert A and B*: Autoradiographic profiles of BaP-DNA adducts formed by BaP activated with rat supersomal CYP1A1 (A) and those with the same enzyme (rat supersomal CYP1A1) but without BaP (B). Values represent mean total RAL (relative adduct labelling) ± SD (*n* = 3; analyses of three independent *in vitro* incubations). ^##^*P <* 0.01 (ANOVA with post-hoc Tukey HSD Test), significant differences between levels of total DNA-BaP adducts formed in incubations with CYP1A1 in the absence and presence of EE2 or estradiol (ESTRA).

In order to resolve the observed discrepancies between the influence of estradiol on formation of BaP-DNA adducts found *in vivo* and in incubations of DNA with BaP using hepatic microsomes *in vitro*, further *in vitro* experiments were conducted using rat recombinant CYP1A1 expressed in Supersomes™ (Fig. 7). The effect of EE2 was also studied in this experimental system. The BaP-DNA adduct pattern obtained by TLC-^32^P-postlabelling in incubations of BaP and DNA using rat supersomal CYP1A1 consisted again of two major spots, namely dG-*N^2^*-BPDE (adduct 1) and adduct 2 derived from 9-hydroxy-BaP-4,5-epoxide (Fig. 7, insert A). In this experimental system, levels of adduct 2 were higher than levels of adduct 1 (*i.e.* dG-*N^2^*-BPDE) (Supplementary Table S2). No BaP-DNA adducts were found in control incubations without BaP (Fig. 7, insert B) or without CYP1A1 or with this enzyme and EE2 and estradiol instead of BaP (data not shown).

The formation of both BaP-DNA adducts was decreased in the CYP1A1-catalysed BaP activation by the presence of EE2 and estradiol (Supplementary Table S2). Total BaP-DNA adduct levels were up to 1.8-fold lower in the presence of EE2 or estradiol (Fig. 7). These findings corresponded to the situation *in vivo* where BaP-DNA adduct formation was reduced in livers of rats treated with BaP in combination with these estrogenic EDs (compare Fig. 3).

## 4 Discussion

The results of the present study emphasise the importance to investigate the effects of EDs when they act in combination. This is also a crucial feature when examining the toxic effects of chemicals present in complex mixtures, because humans are usually exposed to a complex mixture of chemicals which can include both exogenous and endogenous EDs as well as carcinogens. Drug-drug interaction in combination with toxicants may also be critical. However, such studies are rare at the present time. Here, we investigated the combined effects of three EDs, exogenous compounds such as EE2 and BaP as well as estradiol as an example of an endogenous compound, all compounds whose (geno)toxic effects depend on their metabolism. Combined exposure to these chemicals could impact on xenobiotic-metabolising enzymes thereby impacting on their metabolism and genotoxicity (i.e. BaP-DNA adduct formation).

In this study, BaP was found to form covalent BaP-DNA adducts in rat liver. In contrast none of the tested estrogenic compounds (EE2 or estradiol) were capable of generating covalent DNA adducts under the experimental conditions used. However, it should be noted that DNA adduct formation by EE2 and estradiol cannot be fully excluded. Metabolism of estradiol results in the oxidation to semiquinones and quinones that can covalent bind to DNA bases such as deoxyadenosine (Vale et al., 2017) causing apurinic sites (Cavalieri and Rogan, 2016). Nevertheless, those adducts cannot be detected by the ^32^P-postlabelling method used in our study.

However, both EE2 and estradiol influenced the genotoxic properties of BaP, when administered to rats in combination with BaP. We found that the formation of dG-*N^2^*-BPDE adducts in rat liver was decreased when BaP treatment was combined with EE2 or estradiol. We first suggested that the mechanism for the reduced BaP-DNA adduct formation could be linked to a decrease in the levels and activities of CYP enzymes activating BaP, mainly CYP1A1 and 1B1 (Luch and Baird, 2005). However, in the present study we found that estradiol can even increase BaP-induced CYP1A enzyme activity. CYP1A1 and 1B1 are both also capable of metabolising estradiol (reviewed in Zhu and Lee, 2005), whereas EE2 is not metabolised by these CYPs (Borek-Dohalska et al., 2014, Borek-Dohalska et al., 2015). In order to test our hypothesis, we investigated how expression of CYP1A1 and 1B1 is influenced by the combined treatment of rats with BaP and estrogenic EDs, EE2 and estradiol. As expected BaP exposure affected CYP1A1 and 1B1 expression in rat livers differently; we found strong induction of CYP1A1 and moderate induction of CYP1B1, both on the transcriptional and translational level. Because BaP-mediated CYP1A1 induction predominates in this organ and CYP1A1 is more efficient in BaP activation than CYP1B1 (Uppstad et al., 2010; Sulc et al., 2016; Shiizaki et al., 2017), its impact on BaP-DNA adduct formation in rat liver should be more important than CYP1B1. More importantly, BaP-mediated induction of these enzymes was influenced by treatment of rats with EE2 or estradiol together with BaP. In rats treated with BaP/EE2 BaP-mediated induction of CYP1A1 and 1B1 was decreased. Interestingly, co-treatment of rats with BaP together with estradiol also led to the decrease in the BaP-induced *CYP1A1*gene expression, but no changes were found in BaP-enhanced CYP1A1 protein levels, and CYP1A enzyme activity (Sudan I hydroxylation and EROD) was even slightly increased.

The levels of BaP-DNA adducts (dG-*N^2^*-BPDE and the adduct derived from 9-hydroxy-BaP-4,5-epoxide) formed in *in-vitro* incubations with hepatic microsomes isolated from all treatment groups correlated with CYP1A1 enzyme activities in these microsomes (r = 0.959, *P <* 0.01 for Sudan I oxidation and r = 0.997, *P <* 0.01 for EROD) (compare Fig. 4, Fig. 5). This finding demonstrates that BaP-DNA adduct formation in hepatic microsomes is dictated by the CYP1A1 enzyme activities. However, we found differences between the effects of EE2 and estradiol on BaP-DNA adduct formation in microsomal incubations *in vitro* and in rat liver *in vivo*; estradiol lowered BaP-DNA adduct formation *in vivo*, but not in microsomal incubations *in vitro* (compare Fig. 3, Fig. 4).

In order to explain the differences observed for estradiol, we utilised an additional experimental *in-vitro* system. Rat CYP1A1 recombinantly expressed in Supersomes™ was employed to activate BaP to form BaP-DNA adducts and the efficacy of this rat enzyme to form BaP-DNA adducts in the presence of EE2 and estradiol was determined. BaP-DNA adduct formation catalysed by rat CYP1A1 was significantly decreased by EE2 and estradiol in these *in vitro* incubations. Hence, these results corresponded to the situation in rat liver *in vivo*. A decrease in BaP-DNA adduct formation by EE2 and estradiol is probably caused by the inhibition of BaP oxidative activation mediated by CYP1A1, because EE2 and estradiol act as inhibitors of CYP1A1-catalysed EROD activity (Klinger et al., 2002; Chang et al., 2009). Collectively these finding confirm results of former studies demonstrating the predominant role of CYP1A1 in the BaP-DNA adduct formation *in vivo* (Luch and Baird, 2005), they do however not explain the different results found *in vivo* and in microsomal- and CYP1A1-incubations. We can only speculate and several reasons for this phenomenon can be considered: (*i*) BaP-DNA adduct formation in hepatic microsomes might be mediated not only by CYP1A1, but also by some of other CYPs, *i.e.* enzymes of the CYP2C subfamily that metabolise BaP and are expressed in rat hepatic microsomes at high levels. CYP2C form ∼55% of the CYP complement in rat liver (Nedelcheva and Gut, 1994; Večeřa et al., 2011; Zachařová et al., 2012; Stiborová et al., 2015). CYP2C enzymes can be influenced by estradiol in a different way than CYP1A1; for example CYP2C enzyme activities can be stimulated by estradiol or its metabolites; (*ii*) In rats treated with estradiol and BaP, estradiol can be metabolised to metabolites or end products that can be present as residues in hepatic microsomes. These might influence microsomal enzymes by stimulation causing an increase in formation of BaP-DNA adducts; and (*iii*) Several effects of EE2 and estradiol on BaP-DNA adduct formation *in vivo* can also be taken into account. BaP-DNA adduct formation *in vivo* not only depends on the bioactivation of BaP (catalysed by CYPs and/or *m*EH) but also on its detoxification and EE2 and estradiol may also modulate both phase I and phase II enzymes that catalyse BaP detoxification. Moreover, the expression of estrogen-protective enzymes, catechol-*O*-methyltransferase (COMT) and NAD(P)H:quinone oxidoreductase 1 (NQO1), and estrogen-activating enzymes CYP19 and CYP1B1 might influence the genotoxic effects of estrogens (Dawling et al., 2001; Cavalieri and Rogan, 2016). NQO1 is also included into the activation of BaP. Therefore, all these enzymes may also contribute to the different results observed *in vivo* and in microsomal- and CYP1A1-incubations *in vitro* in our study. Further, BaP-DNA adduct formation *in vivo* also depends on the rate of repair of BaP-DNA adducts and EE2- and estradiol-induced gene expression may impact on DNA damage response *in vivo*. However, all these suggestions should be further investigated but were beyond the scope of the present study.

Our present results demonstrate not only the importance of studying mixtures of xenobiotics, but they also illustrate an experimental approach how such studies should be carried out when investigating the metabolism and genotoxic properties of EDs or toxicants generally mediated by biotransformation enzymes (*i.e.* CYPs). A combination of *in vivo* and *in vitro* experiments is one of the essential approaches to be performed. Furthermore, care has to be taken when selecting the enzymatic *in vitro* systems used. In many studies pure enzymes or subcellular microsomal fractions are frequently employed separately, but should be used in combination as illustrated here. We also recommend performing correlation analyses of data found in experimental approaches to better relate *in vivo* and *in vitro* findings.

## 5 Conclusion

We found that both EE2 and estradiol have an effect on BaP-DNA adduct formation and on CYP1A1 expression, the enzyme predominantly catalysing this process in rats *in vivo* when rats are exposed to these estrogens in combination with BaP. This suggests that EE2 and estradiol may share a common pathway which influences CYP1A1 expression thereby modulating BaP bioactivation. Moreover, BaP could also influence CYP1A1-mediated estradiol/EE2 genotoxicity. Although not analysed in the present study estradiol is known to be capable of forming DNA adducts and other CYP enzymes have been shown to catalyse this reaction (Vale et al., 2017). Our results demonstrate the importance of studying mixtures of BaP with estrogenic compounds, and they also illustrate an approach how such studies can be carried out. Combining both *in vivo* and *in vitro* experiments is one of the essential approaches to be performed as illustrated in other studies investigating BaP metabolism (Arlt et al., 2008; Reed et al., 2018). Overall our study provides evidence that more consideration should be given to potential ED-ED interactions when humans are exposed to these toxicants.
