# Supplementary material for: Exposure to endocrine disruptors 17alpha-ethinylestradiol and estradiol influences cytochrome P450 1A1-mediated genotoxicity of benzo[a]pyrene and expression of this enzyme in rats
Source: Toxicology. 2018 May 1;400-401:48–56. doi: 10.1016/j.tox.2018.04.001 (PMC6593260; doi:10.1016/j.tox.2018.04.001)
Supplement: Supplementary file 1 [file mmc1.docx]

**Exposure to endocrine disruptors 17alpha-ethinylestradiol and estradiol influences cytochrome P450 1A1-mediated genotoxicity of benzo[*a*]pyrene and expression of this enzyme in rats**

**Marie Stiborová^a,*^, Helena Dračínská^a^, Lucie Bořek-Dohalská^a^, Zuzana Klusoňová^a^, Jana Holecová^a^, Markéta Martínková^a^, Heinz H. Schmeiser^b^, Volker M. Arlt^c^**

*^a^ Department of Biochemistry, Faculty of Science, Charles University, Albertov 2030, 128 40 Prague 2, Czech Republic*

*^b^ Division of Radiopharmaceutical Chemistry, German Cancer Research Center (DKFZ), Im Neuenheimer Feld 280, 69120 Heidelberg, Germany*

*^c^ Department of Analytical, Environmental and Forensic Sciences, MRC-PHE Centre for Environment and Health, King’s College London, 150 Stamford Street, London SE1 9NH, United Kingdom*

^*^**Corresponding author at**: Department of Biochemistry, Faculty of Science, Charles University, Albertov 2030, 128 40 Prague 2, Czech Republic.

*E-mail address*: [stiborov@natur.cuni.cz](mailto:stiborov@natur.cuni.cz) (M. Stiborová).

**Supplementary tables**

**Supplementary Table 1**

DNA adduct formation by BaP, measured by TLC-^32^P-postlabelling, activated with hepatic microsomes isolated from liver of rats exposed to BaP, EE2 or estradiol (ESTRA) alone and in combination (BaP+EE2, BaP+ESTRA or EE2+ESTRA). Values represent mean RAL (relative adduct labelling) of adducts 1 and 2 ± SD (*n*=3; analyses of three independent *in vitro* incubations). ^**^*P*<0.01 (ANOVA with post-hoc Tukey HSD Test), significant differences between levels of DNA-BaP adducts 1 and 2 formed in incubations with hepatic microsomes of rats treated with BaP alone and combinations of BaP with EE2 and estradiol (ESTRA).

|  | **RAL/10^6^ nucleotides** | |
| --- | --- | --- |
|  | **dG-*N*^2^-BPDE adduct**  (adduct 1) | **9-OH-BaP-4,5-epoxide adduct**  (adduct 2) |
| **control** | N.D. | 0.219 ± 0.067 |
| **BaP** | 2.614 ± 0.961 | 15.461 ± 0.853 |
| **EE2** | N.D. | 0.291 ± 0.085 |
| **ESTRA** | 0.079 ± 0.041 | 0.202 ± 0.045 |
| **BaP+EE2** | 5.307 ± 0.409 ** | 9.348 ± 0.559 ** |
| **BaP+ESTRA** | 9.961 ± 1.053 ** | 25.382 ± 2.885 ** |
| **EE2+ESTRA** | 0.227 ± 0.049 | 0.521 ± 0.104 |

N.D., Not detected.

**Supplementary Table 2**

DNA adduct formation by BaP, measured byTLC-^32^P-postlabelling, activated with rat CYP1A1 *in vitro* and the effect of EE2 and estradiol (ESTRA) on the levels of adducts. Values represent mean RAL (relative adduct labelling) of adducts 1 and 2 ± SD (*n*=3; analyses of three independent *in vitro* incubations). ^**^*P*<0.01, ^*^*P*<0.05 (ANOVA with post-hoc Tukey HSD Test), significant differences between levels of DNA-BaP adducts 1 and 2 formed in incubations with CYP1A1 in the absence and presence of EE2 or estradiol (ESTRA).

|  | **RAL/10^7^ nucleotides** | |
| --- | --- | --- |
|  | **dG-*N*^2^-BPDE adduct**  (adduct 1) | **9-OH-BaP-4,5-epoxide adduct**  (adduct 2) |
| **BaP** | 19.14 ± 5.08 | 61.49 ± 7.57 |
| **BaP+EE2** | 10.44 ± 2.31 * | 35.00 ± 6.45 ** |
| **BaP+ESTRA** | 10.60 ± 3.88 * | 35.88 ± 6.17 ** |
